# Predicting and Validating Protein Interactions Using Network Structure

**DOI:** 10.1371/journal.pcbi.1000118

**Published:** 2008-07-25

**Authors:** Pao-Yang Chen, Charlotte M. Deane, Gesine Reinert

**Affiliations:** Department of Statistics, University of Oxford, Oxford, United Kingdom; Columbia University, United States of America

## Abstract

Protein interactions play a vital part in the function of a cell. As experimental techniques for detection and validation of protein interactions are time consuming, there is a need for computational methods for this task. Protein interactions appear to form a network with a relatively high degree of local clustering. In this paper we exploit this clustering by suggesting a score based on triplets of observed protein interactions. The score utilises both protein characteristics and network properties. Our score based on triplets is shown to complement existing techniques for predicting protein interactions, outperforming them on data sets which display a high degree of clustering. The predicted interactions score highly against test measures for accuracy. Compared to a similar score derived from pairwise interactions only, the triplet score displays higher sensitivity and specificity. By looking at specific examples, we show how an experimental set of interactions can be enriched and validated. As part of this work we also examine the effect of different prior databases upon the accuracy of prediction and find that the interactions from the same kingdom give better results than from across kingdoms, suggesting that there may be fundamental differences between the networks. These results all emphasize that network structure is important and helps in the accurate prediction of protein interactions. The protein interaction data set and the program used in our analysis, and a list of predictions and validations, are available at http://www.stats.ox.ac.uk/bioinfo/resources/PredictingInteractions.

## Author Summary

For understanding the complex activities within an organism, a complete and error-free network of protein interactions which occur in the organism would be a significant step forward. The large amount of experimentally derived data now available has provided us with a chance to study the complicated behaviour of protein interactions. The power of such studies, however, has been limited due to the high false positive and false negative rates in the datasets. We propose a network-based method, taking advantage of the tendency of clustering in protein interaction networks, to validate experimental data and to predict unknown interactions. The integration of multiple protein characteristics (i.e., structure, function, etc.) allows our predictive method to significantly outperform two other approaches based on homology and protein-domain relationships on datasets which contain a large amount of interactions, but not much detailed information on the proteins involved in the interactions. In addition, our predictive score based on triadic interaction patterns improves over a pair-wise approach, suggesting the importance of network structure. Moreover, using pooled interactions as prior information, we find evidence for fundamental differences in protein interaction networks between eukaryotes and prokaryotes.

## Introduction

For understanding the complex activities within an organism, a complete and error-free network of protein interactions which occur in the organism would be a significant step forward. Experimentally, protein interactions can be detected by a number of techniques, and the data is publicly available from several databases such as DIP, Database of Interacting Proteins [1], and MIPS, Munich Information Center for Protein Sequences [2]. Unfortunately, these experimentally detected interactions show high false negative [3] and high false positive rates [4],[5]. In this paper we develop a new computational approach to predict interactions and validate experimental data.

Computational methods have already been developed for these purposes. For interaction validation, these have mainly centered on the use of expression data [5],[6] or the co-functionality or co-localisation of the proteins involved [7],[8].

For prediction of protein interactions in contrast, many methods have been suggested. The majority of these generate lists of proteins with a functional relationship rather than physical interactions [9],[10].

In terms of physical interaction prediction the available methods can be typified by the two approaches of Deng et al. [11] and Jonsson et al. [12].

In Deng et al.'s method, a domain interaction based approach, a protein interaction is inferred on the basis of domain contacts. If a domain pair is frequently found in observed protein interactions, it is likely that other protein pairs containing this domain pair might also interact. From the observed protein interaction network, the probabilities of domain-domain interactions are estimated. The expectation-maximum algorithm is employed to compute maximum likelihood estimates, assuming that protein interactions occur independently of each other. This likelihood is then used to construct a probability score for a protein pair to interact, it is inferred based on the estimated probabilities of domain interactions within the protein pair. Deng et al.'s prediction is based on a total of 5,719 interactions from *S.cerevisiae*. However, the limited number of known domains may well not be enough to describe the variety of protein interactions. This approach has had further extensions, such as an improved scoring for domain interactions [13] and the inclusion of other biological information [14]. Liu et al.'s model [15] is an extension of Deng et al.'s method which integrates multiple organisms. In addition to *S.cerevisiae*, two other organisms, *C.elegans*, *D.melanogaster*, are included.

The second type of approach, as used by Jonsson et al. [12], is homology-based. It searches for interlogs among protein interactions from other organisms. If an interlog of a protein interaction exists in many other organisms, this protein interaction will score highly. In addition to searching for orthologous interlogs, Mika and Saeed [16],[17] suggest that paralogous interlogs may provide even more information for inferring interacting protein pairs.

In principle, statistical clustering algorithms such as [18] and [19] which identify cliques in the network could be viewed as a prediction method, predicting that all proteins within a clique interact with each other. This interpretation is biologically questionable, and as the focus in the statistical clustering approach is on locating cliques and overlapping modules rather than on predicting individual interactions, we exclude it from our comparisons.

Neither Deng et al.'s method nor Jonsson et al.'s method make use of network structure beyond pairwise interactions; interactions are considered as isolated pairs. However these pairs could and should be considered as a network, where the proteins are nodes and their interactions are links [20],[21]. Topological examination of these networks has revealed many interesting properties, including a clustering tendency [22],[23], see also Supporting Information (Text S1, Table S1). In our method we exploit the network structure by developing a score which considers triadic patterns of interactions rather than pairs. In this paper we thus take the established idea that the characteristics of a protein (i.e., its structure, function and location) will affect its interactions (see for example [7], [21], [24]–[31]) alongside the not yet fully explored idea that its network position will also affect its interactions, in order to develop a novel predictive tool.

Our goal is to predict (undirected) protein interactions of the type *x* with *y*, where both *x* and *y* interact with a third protein *z*. Therefore in our approach we particularly focus on two simple three node network structures, triangles and lines. A *triangle* is a subnet formed by an interacting protein pair with a common neighbour. A *line*, by contrast, is a subnet formed by an non-interacting protein pair with a common neighbour. We will show that these network structures and the protein characteristics within them help to predict protein interactions.

We apply our method to the *S.cerevisiae* interaction network from the DIP database. During the validation we assume that function and structure are known for all proteins (fully annotated) and that the protein interaction network is known for all but one interaction. With triadic interacting patterns, we predict the interaction status of those protein pairs with at least one common neighbour and compare our results with those from three other published scores. We go on to demonstrate that the requirement to have fully annotated proteins can be relaxed to include partially annotated proteins, with a slight drop in the accuracy. The prediction is also compared with simulated networks where all proteins are shuffled while the network structure is maintained, in order to examine whether the specific network structure, triangles and lines, keep useful information in forming protein interaction networks.

To measure the true positive rate in a set of protein pairs, Deane et al [5] proposed the expression profile index (EPR), a measure of the true positive rate in a set of protein pairs based on biological relevance. We compare the EPR index to our score, showing that, with a suitable cut-off, our predictions achieve a high true positive rate. We also give examples of validated experimental data and predict new interactions.

Our predictive model uses a prior interaction database and for this we use three prior databases, pooling protein interactions collected from prokaryotes, eukaryotes and all interactions. The results from using different prior databases show that the use of interactions from within the same kingdom rather than across kingdoms significantly improves the results, indicating as in [21] that interaction networks may be significantly different between the kingdoms.

Comparing our method to three other standard approaches, namely the domain-based approach by Deng et al. and an extension by Liu et al., and a homology-based approach by Jonsson et al., we find that our method outperforms the above approaches on the subset of interactions in the DIP Yeast data set which contains enough annotation and connectivity to be included in our analysis. Our method complements the methods by Deng et al. and Liu et al., as their approaches apply to a rather different subset of potential interactions yielded from the DIP Yeast data set.

## Materials and Methods

### Protein Interaction Networks

Experimental protein interactions of *S.cerevisiae*, excluding self-interactions, are obtained from DIP (DIP Yeast). Self-interactions (<3% of all interactions) are excluded, implying that all triangles and lines are constructed of three different proteins. Three different prior data bases are constructed by pooling interactions considering eukaryotes (*D.melanogaster*, *C.elegans*, *S.cerevisiae*, *M.musculus*, *H.sapiens*), prokaryotes (*E.coli* and *H.pylori*), or all interactions; the interaction we would like to predict or to validate is always excluded.

### Classifications of Structure and Function

The proteins in our dataset are classified into the seven SCOP classes [32] using the SUPERFAMILY database [33], see Supporting Information (Text S1, Table S3). Between 61 to 89% of proteins are classified, dependent on organism. In our analysis, a protein is found to be assigned to 1.3 classes on average.

We use the 24 functional groups from the secondary level of Molecular Function in the Gene Ontology [34], see Supporting Information (Text S1, Table S4) as our protein functional categorisation. Molecular Function ontology in GO has 188 secondary level categories, excluding the categories “obsolete” and “unknown”. The 24 groups used are those that are most frequently observed. An annotated protein may be assigned to several nodes in GO, which can be traced back to one or multiple nodes.

### The Upcast Sets of Characteristic Triplets

**Figure 1:**
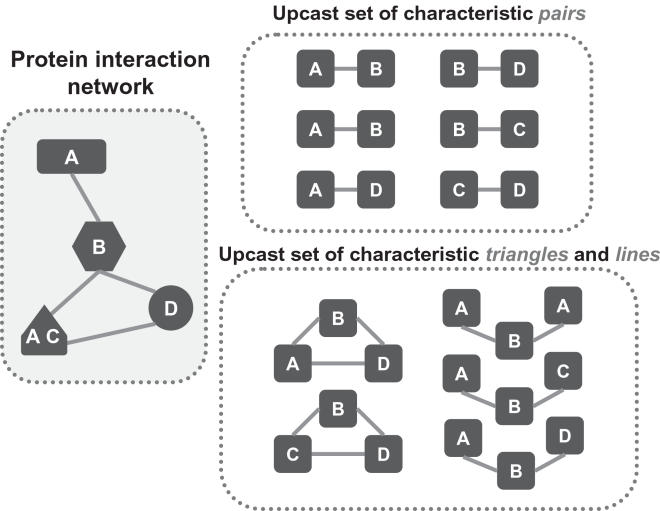
Upcast Sets of Characteristic Pairs and Triplets. In this example, we consider only a single characteristic (e.g., protein function), so that the characteristic vector for a protein is a 1-vector. There are three single-category proteins and one two-category protein in the protein interaction network (left), which result in an upcast set of six characteristic pairs {*A*–*B*, *A*–*B*, *A*–*D*, *B*–*D*, *B*–*C*, *C*–*D*}. Alternatively, the upcast set of triplets includes two triangles and three lines.

The protein interaction network is used to build an upcast set of triplets of characteristic vectors as in Figure 1; see also [21]. Here, *A*, *B*, *C* and *D* denote protein characteristics, whereas different shapes indicate different proteins. A protein may possess more than one characteristic. Our triplets are triangles and lines of three characteristic vectors according to their interacting patterns. A characteristic line is a specific pattern constructed by three vectors with two vector interactions among them. A characteristic triangle is formed by three vectors interacting with each other.

Here we abuse the English language; while it would be clearer to say “pair of characteristics” and “triangle of characteristics” we prefer the shorter version “characteristic pair” and “characteristic triangle” for easier reading.

### The Upcast Sets of Characteristic Pairs

To assess our method we also compare it with a score based on characteristic pairs only. In a similar manner to the upcast set of characteristic triplets, we construct an upcast set of characteristic pairs. Here we grasp the opportunity to introduce some notation. For a protein *x*, its characteristic vector *S_c_* (*x*) contains all its characteristics of a certain type (e.g., structure, function), and *S(x)* denotes the set of vectors formed using different characteristics. In the case of two protein characteristics, *S*_1_ (*x*) and *S*_2_ (*x*) are the two respective vectors, and *S*(*x*) is the set
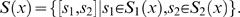



We shall denote the set of all characteristic vectors for all proteins by *S*; this set may contain a vector *v_a_* multiple times.

A *characteristic pair* is constructed by two characteristic vectors from two interacting proteins. If two proteins *x* and *y* interact, for each pair {*ν_a_*, *ν_b_*} with *ν_a_* ∈ *S*(*x*), *ν_b_* ∈ *S*(*y*), we write *ν_a_*∼*ν_b_*. If two protein do not interact, the relation between two vectors is denoted by *υ_a_*≁*υ_b_*. The upcast set of characteristic pairs is then the collection of all characteristic pairs extracted from the protein interaction network, which may stem from one or from multiple organisms.

### Eligible Interactions

For our upcast sets to be informative for a protein interaction, an eligible protein pair has to satisfy two conditions: Firstly, the proteins need to have at least one common interacting neighbour; and secondly, the query protein pair and the neighbours have to be at least partially annotated.

Among 4,931 proteins in the observed interaction network, 2,416 (49%) proteins are fully annotated with both characteristics (structure and function) and 3,808 (77%) are annotated with at least one characteristic.

Table 1 gives the number of eligible protein pairs in the Yeast protein interaction network. There are about 90,000 eligible fully annotated proteins pairs and around 3% of them are in the experimental data (DIP Yeast). When partially annotated proteins are included, the number of eligible protein pairs is increased by 158%.

**Table 1:** The Size of Predictable Protein Pairs in Yeast.

DIP Yeast network	Proteins	Interactions (Percentage)
Observed network	4,931	17,471
fully annotated (F)	2,416	6,537 (37%)
fully and partially annotated (F+P)	3,808	13,102 (75%)
Eligible protein pairs (F)†		87,181
observed interactions		2,896 (3%)
unobserved interactions		84,285 (97%)
Eligible protein pairs (F+P)‡		224,631
observed interactions		6,252 (3%)
unobserved interactions		224,631 (97%)

**†:** proteins annotated with both characteristics (structure and function).

**‡:** proteins annotated with at least one characteristic.

### The Triangle Rate Score

We derive our *triangle rate score* from the upcast sets of characteristic triplets. This score thus includes information not only from the query protein pair but also from its neighbours. Therefore, it is a network-based score which goes beyond pairwise interactions.

Within the triplet interactions, we assess the odds to observe triangles versus lines around the query protein pair. More formally, let *t_xy_* be the total frequency of all characteristic triangles around the query protein pair {*x,y*}; denoting by *z* ∈ *B*(*x,y*) the set of all common neighbours of *x* and *y* in the protein interaction network,
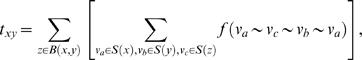
Where *f* (*ν_a_*∼*ν_c_*∼*ν_b_*∼*ν_a_*) is the frequency of triangle {*ν_a_*∼*ν_c_*∼*ν_b_*∼*ν_a_*} among all characteristic triangles in the prior data base. Similarly, *l_xy_* is the total frequency of all characteristic lines around the query protein pair {*x*, *y*}. We define the *triangle rate score*, *tri*(*x,y*) for the protein pair {*x*, *y*}. as the odds of observing triangles versus lines among triangles and lines in its neighbourhood,
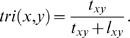
(1)Heuristically, the higher the triangle rate score is, the higher the chance one would observe an interaction between the query protein pair.

When multiple characteristics are simultaneously included, the triangle rate score defined above requires the query protein pair and the common neighbour to be fully annotated with multiple characteristics. However, there are many partially annotated proteins in the neighbourhood which may provide useful information. These proteins are particularly important when only a few fully annotated ones are available. In Supporting Information (Text S1, C), an extended version of the triangle rate score is provided to include partially annotated proteins.

### The Pair-Based Score

To assess whether the triangle rate score significantly improves prediction and validation, we also construct a similar score based on pairwise interactions only, which we call the *pair-based score*. The details are as follows.

Based on the pairwise interactions, we also provide an odds ratio-based score, see also [23] for details, which gives a measure of the relative count of the characteristic pair found between positive and negative protein interactions. We call an interaction “positive”, if it is contained in the database. All potential interactions which are not found in the database are called “negative”. This score can be viewed as a likelihood for a model which assumes that

1. The number of proteins in each type of characteristic vector is multinomially distributed.
2. Given the total number of characteristic pairs which can be derived from the frequency of characteristic vectors, the number of actual interactions for each type of characteristic pair {*ν_a_*, *ν_b_*} is binomially distributed, with the probability of success *π_ab_* being the probability of interaction between the proteins in the pair, and these binomial random variables are independent.

Given a specific characteristic pair {*ν_a_*, *ν_b_*}, under the multinomial-binomial model above the maximum likelihood estimate for *π_ab_* is given by
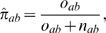
where *o_ab_* is the number of times an interaction has been observed for the characteristic pair {*ν_a_*, *ν_b_*}, and *n_ab_* is the number of times that no interaction was observed for the pair {*ν_a_*, *ν_b_*}.

With this heuristic we define the *pair-based score* for a query protein pair {*x*, *y*} as
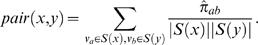
(2)Thus *pair*(*x,y*) is the average of the estimated probabilities *πˆ* *_ab_* for all characteristic pairs generated by the query protein pair in the prior data base. Heuristically, the higher the score, the more likely it should be that the two query proteins interact. An extended version of the score is able to cover protein pairs which are only partially annotated, see Supporting Information (Text S1, C).

We note that the triangle rate score and the pair-based score have a slightly different form. While the pair score is the average of all relative frequencies of characteristic pairs, the triangle rate score is the summed frequency of the characteristic triangles over triangles and lines. The different setting here was chosen because around a query protein pair many characteristic triangles might hardly be seen in the observed networks; their counts are too small to be useful. This phenomenon is much less pronounced for the pair patterns, there being rather more triangle patterns than pair patterns; see Supporting Information (Text S1, Table S2) for the number of observed patterns against all possible patterns.

### The Receiver Operating Characteristic (ROC) Curve

In order to put our scores to work we choose a threshold; all pairs with scores above that threshold would be classified as interacting, while all pairs below that threshold would be classified as non-interacting. The choice of threshold depends on the desired sensitivity and specificity; recall that the *sensitivity* is the ratio of true positives over (true positives+false negatives) and the *specificity* is the ratio of true negatives over (true negatives+false positives). To assess our scores we first use a Receiver Operating Characteristic (ROC) curve, which is a useful technique for examining the performance of a classifier [35]; in our case the classes are “interacting” or “non-interacting” for a pair of proteins. The curve plots sensitivity against (1 minus specificity). Each point on a ROC curve is generated by selecting a score threshold for a method. We move the cutoff along the range of the score and record different sensitivities and specificities of a method. The closer the curve is to the upper left hand corner (i.e., the larger the area under curve), indicating that sensitivity and specificity are both high, the better the predictive score.

#### Validation procedure

While we are never completely certain that a prediction is correct, we assume that a positive prediction is correct if it is contained in our gold-standard positive (GSP) set, and that a negative prediction is correct if it is contained in our gold-standard negative (GSN) set. The GSP set is based on 8,250 hand-curated interactions in MIPS complexes catalog [2],[7]. These positive interactions are identified if two proteins are within the same complex and if the interactions are confirmed by various experimental techniques. The GSP we use is the overlapping protein pairs between our eligible protein pairs described in Table 1 and MIPS complexes catalog. For the comparison between methods we use the overlap between the eligible protein pairs for the respective methods, and the gold standard MIPS set.

The set of gold-standard negatives (GSN) are random protein pairs which neither share protein localisation, nor expression nor homologous interaction data [17].

We have many more gold-standard negatives than positives. The unequal sizes of gold-standard sets may affect the ROC curve; when the cutoff is high, too many gold-standard negatives would cause a rapid increase in true negatives, which would result in artificially high specificity. To avoid this bias, we collect 300 samples of randomly selected pairs from the extensive GSN. Each sample is the same size as our GSP set. Predictions are verified against these 300 reference sets obtained by combining the GSP set and the sample from the GSN set.

#### Testing difference between two ROC curves

In order to differentiate the ROC curves of the different predictors we have developed a method to compare the areas under two curves (AUC) [35],^36^ through the statistical *z*-test for differences. Since the AUC is limited by a unit square, its value will be between 0 and 1.0. While there is a possibility for a correlation between the AUC of two samples, randomly generating 1,000 samples of two sets of 30 random samples from the set of 300 AUC values, no significant covariance was detected for any of the scores under consideration. Hence assuming that our 300 samples are approximately independent, from the Central Limit Theorem the average AUC should be approximately normally distributed. Therefore here we used a *z*-test to compare the mean difference between the 300 AUC from two scores. If the difference between two mean AUC is too large then we reject the null hypothesis that two AUC are equal and conclude that there is evidence that one ROC curve is significantly better than the other one. Here we not only use tests at 5% significance level; but we also give the *p*-values of the tests. For details of the *z*-test see Supporting Information (Text S1, D).

### The Precision-Recall Operating Characteristic (P-ROC) Curve

When evaluating performance for a classifier when the test data is unbalanced, such as when there is a disproportionate number of negative versus positive cases, instead of choosing subsamples of the same size as for our tests between two ROC curves, the Precision-Recall Operating Characteristic (P-ROC) curve provides an alternative. The *precision* is the ratio of true positives over (true positives+false positives), whereas the *recall* is the ratio of true positives over (true positives+false negatives), i.e. the sensitivity. The P-ROC curve plots recall against precision. While there is a tendency for recall and precision to be inversely related, Precision-Recall curves are not necessarily decreasing. An increasing P-ROC curve is an indication for *perverse retrieval*, in which there is a strong tendency that first the negative interactions are retrieved; only when there are so few of those left that it is almost unavoidable to retrieve positive interactions, these are also covered; see for example [37] for an exposition.

## Results/Discussion

Initially we compare our method to the methods suggested by Deng et al. [11], Liu et al. [15], and Jonsson et al. [12], we then compare it to our pair-based variant. All these comparisons are carried out using a leave-one-out cross validation approach where one eligible protein pair is excluded from the Yeast network prior database. Finally we establish the power of the method when partially annotated proteins are included in the process.

### Comparison with Other Published Methods

We compare our triangle rate score with three other methods, the two by Deng et al. [11] and Liu et al. [15] being domain-based, and the one Jonsson et al. [12] being homology-based. The two scores by Deng et al. and Liu et al. are downloaded directly from the authors' webpages. Deng et al.'s method predicted 125,435 protein pairs. After removal of 5,717 interactions, which are the training data in forming the scores, and translating the gene names to ORF names (to match the reference sets), 63,013 protein pairs remained. Liu et al.'s method predicted 20,088 protein pairs. After the translation of names, 15,608 protein pairs remained. Our triangle rate score predicts 87,181 protein pairs. The number of predicted pairs using the different methods on the DIP 20060402 data set described above, and the overlap with our pairs, given in Table 2, illustrates that our method and Deng et al. and Liu et al.'s methods complement each other, as they operate on fairly disjoint sets. In contrast, there is a substantial overlap between the eligible pairs for Jonsson et al.'s score and for the triangle score.

**Table 2:** Eligible Protein Interactions for Different Methods.

Method	No. eligible pairs	Overlap with eligible triangle rate score pairs
Deng et al.'s score	63,013	2,950
Liu et al.'s score	15,608	746
Jonsson et al.'s score	59,039	38,231

**Figure 2:**
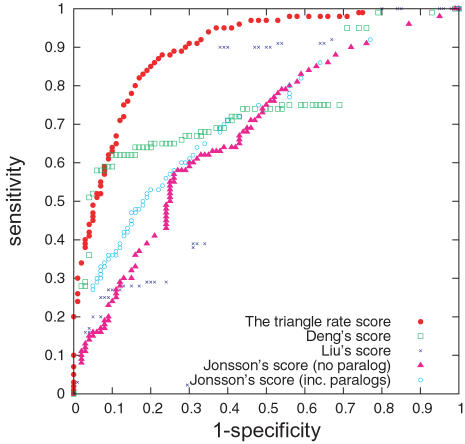
ROC Curves of Predictive Scores. The ROC curves, 1 minus specificity vs. sensitivity, for predicting yeast protein interactions using domain interaction based approaches (Deng et al.'s score and Liu et al.'s score), a homology-based approach (Jonsson et al.'s score plus paralogs) and our network-based approach (the triangle rate score).

Jonsson et al.'s method is implemented in two ways, using orthologs only (a pooled database of 6 organisms, *E. coli*, *H. pylori*, *C. elegans*, *D. melanogaster*, *M. musculus* and *H.sapiens* from DIP for the search of similar sequences), and additionally using orthologs and paralogs (see Figure 2 and Table 3). In the second case the *S. cerevisiae* interactions in DIP are also included.

**Table 3:** Areas under ROC Curves for Scores Comparison.

Predictive scores	Mean (*x̅*)	Sample standard deviation (*s*_x̅_)
The pair-based score	0.841	0.0066
The triangle rate score	0.893	0.0058
Deng et al.'s score	0.757	0.0191
Liu et al.'s score	0.705	0.0228
Jonsson et al.'s score	0.677	0.0135
Jonsson et al.'s score (inc. paralogs)	0.712	0.0084

The comparison of scores are shown in Figure 2. The areas under the ROC curve were tested for significant difference; see Table 3. The results of the *z*-tests show that our triangle rate score outperforms both the domain-based (second place) and homology-based scores, see Table 4 for *p*-values. Here the comparison with the domain-based methods has to be taken with a pinch of salt, as the amount of overlap between the eligible pairs for those methods and our method is very small.

**Table 4:** Z-tests for AUC Comparison among Predictive Scores.

Predictive scores	P	T	D	L	J	JP
The pair-based score (P)		*	*	*	*	*
The triangle rate score (T)			*	*	*	*
Deng et al.'s score (D)				0.079	*	0.031
Liu et al.'s score (L)					0.281	0.770
Jonsson et al.'s score (J)						0.025
Jonsson et al.'s score (inc. paralogs) (JP)						

*: : *z*-score>3.29, i.e., *p*-value<0.001.

**Figure 3:**
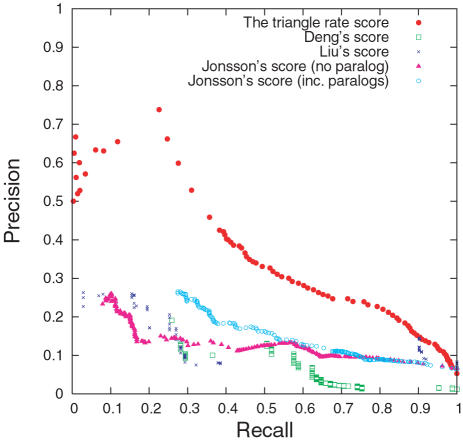
P-ROC Curves for Comparison among Scores. The P-ROC curves for the comparison of scores.

The P-ROC curve in Figure 3 for the comparison between the different methods shows not only that the triangle rate score outperforms the other methods on our data set, but it also reveals that Deng et al.'s score and Liu et al.'s score have marked jumps in recall. The overlap with our data set is so small that these jumps may be artefacts.

The number of predictions which overlap with the MIPS-GSP (8,250 interactions) is also an indicator of coverage. Our triangle rate score is able to predict 928 of them, which is the largest number of predictions from any of the four sets. Deng et al. and Liu et al.'s scores, based on protein-domain relationships, can only predict 85 and 174 interactions in GSP respectively. Their methods cannot predict protein pairs without domain information, limiting their coverage. Liu et al.'s score, when including information from other organisms, improves the coverage over Deng et al.'s score, but not the overall performance in terms of AUC. Jonsson et al.'s score covers more interactions in GSP (390 interactions) than the domain interaction based approaches, however, it appears to perform worse in terms of AUC, though not significantly. Jonsson et al.'s method is still limited in coverage, however, because only sequences with very high similarity are useful for transferring interactions, and often qualified homologs are not available, see [16].

### Comparison between the Triangle Rate Score and the Pair-Based Score

**Figure 4:**
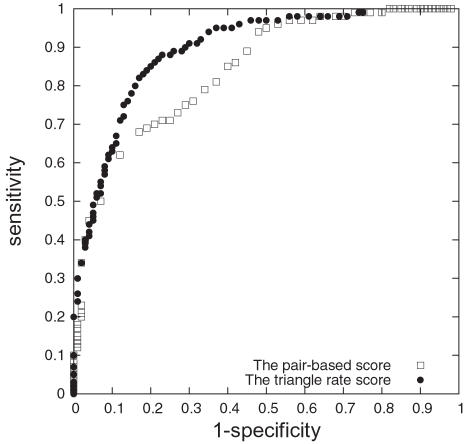
ROC Curves of Pair-Based Score and Triangle Rate Score. The ROC curves for interactions prediction from the triangle rate score and the pair-based score.

We also compare our triangle rate score to the pair-based score, thus allowing us to ascertain the effect of network structure on our scoring method. The ROC curves in Figure 4 show that the triangle rate score outperforms its pair-based analog, thus demonstrating that the inclusion of network information beyond pairwise interactions significantly improves prediction. The success of the triangle rate score indicates the importance of network structure (triangles and lines) in conjunction with protein characteristics for the understanding of protein interactions.

We have also employed a logistic regression model to include pair- and triplet-based statistics, see Supporting Information (Text S1, E) for details. As the preliminary investigation did not show significant improvement over the simple triangle rate score and the full scale leave-one-out validation would be very computation-expensive we did not pursue this model further.

### The Performance of the Triangle Rate Score

The triangle rate score can be used to validate experimentally derived interactions. It is estimated that the false positive rates for high-throughput experiments vary from 35 to 83% dependent on source [3].

At a cut-off score value of 0.09, our prediction reaches 0.83 for both sensitivity and the specificity. Of the 2,896 DIP Yeast interactions tested by the triangle rate score, 1,732 (60%) are validated at the score cut off of 0.09. This gives an estimated false positive rate of around 40%, close to that given by EPR [5].

**Figure 5:**
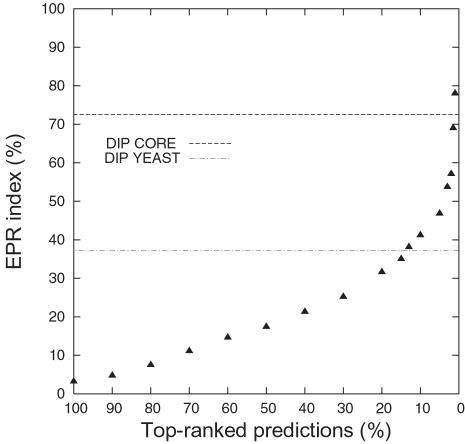
EPR Index in Predictions of Interactions. The black triangles indicate the EPR index for the predicted interactions for top-ranked scores. For example, the set of top 10% predictions has EPR index 41.2.

We also calculate the EPR index (% correct) for subsets of our predictions. Figure 5 shows how the EPR index increases with higher ranked prediction sets. As our score cut-off is increased, the EPR index indicates that the quality of our predictions is increasing. The set of the top 14% predictions (∼12,200 interactions) shows a higher EPR than the experimentally derived interactions in DIP Yeast.

The EPR index estimates the biologically relevant fraction of protein interactions detected in a high throughput screen. As the EPR index is between 70–80% for DIP CORE, we cannot hope for a correct prediction rate (fraction of true predictions over true positives) higher than 70–80%. Indeed this upper limit is reflected by a sharp drop-off in the ROC curve (Figure 2) for (1- specificity) between 0.2 and 0.3, i.e. specificity between 0.7 and 0.8.

**Figure 6:**
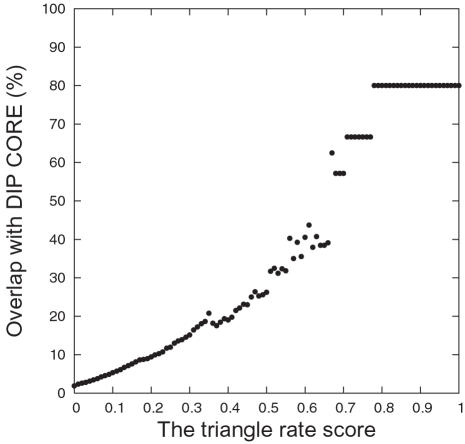
Percentages of Predictions of Interactions Overlapping with DIP CORE. For each triangle rate score, the amount of overlap of predicted interactions (score≥this rate) with DIP CORE is plotted.

A second way to assess the accuracy of our predicted set is to consider the overlap between our positive predictions and DIP CORE. DIP CORE includes 5,969 high-confidence interactions determined by one or more small scale experiments. As shown in Figure 6, the percentage of overlap increases with increasing score cut-off values. Both these tests demonstrate that the triangle rate score is a good indicator of interaction prediction quality.

#### Prediction of new interactions using the triangle rate score

To assess how our triangle rate score predicts in practice, we look at the 652 protein pairs with high triangle rate scores (the top 1%; ∼871 pairs) that are not observed in DIP Yeast. Among these pairs, about 80% are co-localised and 60% share the same function. Indeed, half of them share both function and subcellular location. These findings indicate that such highly scoring protein pairs are likely interactors.

Among five randomly chosen pairs, two were confirmed by manually checking other public protein databases such as BioGRID [38] and YPD [39], and literature databases such as Pubmed. These two cases are described below.

The two proteins “ATP synthase subunit 4” (YPL078C) and “ATP synthase subunit epsilon” (YPL271W) are both in the DIP database, but DIP does not record an interaction between them. The pair receives a very high triangle rate score, suggesting a possible interaction. From BioGRID and Pubmed, we find that their interaction is confirmed in a co-purification experiment and in the literature [40]. These two proteins are part of units for mitochondrial ATP synthesis and they both belong to a large evolutionarily conserved enzyme complex.

Our second example is the pair “Transcription initiation factor TFIID subunit 1” (YGR274C) and “Transcription initiation factor IIA small subunit” (YKL058W), which also has a high triangle rate score. Both share transcriptional activation as on of their functions; their positive interaction can be verified in the literature in [41] and BioGRID.

#### Validation of experimental interactions using the triangle rate score

We can also consider the converse, using the triangle rate score to validate a stated interaction, with the aim to identify potentially false positives. We examined our lowest scoring 5% (4,355 protein pairs); 49 of which are found in DIP Yeast. Among these 49 pairs, 42 do not share the same function. There are 11 pairs that share neither function nor subcellular location. One example is the interaction between “Protein TEM1” (TEM1) and “Long-chain-fatty-acid–CoA ligase 4” (FAA4). The database entry is based on Yeast two-hybrid experiments, a particularly error-prone experimental technique. While TEM1 is located in cytoskeleton, endoplasmic reticulum, or punctate composite, FAA4 is in cytoplasm. In terms of functional categories, TEM1 involves in nucleotide binding and in hydrolase activity, and FAA4 is in long-chain-fatty-acid-CoA ligase activity. These two proteins are located differently and share no common function, raising a question mark on whether they indeed interact. False positive interactions could arise from several reasons, such as autoactivation of reporter transcription by the bait protein alone. We suggest that a small-scale experiment should be carried out on this specific protein pair.

A list of the high scoring protein pairs which are not in DIP and a list of low scoring pairs which are in DIP are provided in Supplementary Information Dataset S1 and Dataset S2, respectively.

### Including Partially Annotated Proteins

The triangle rate score can be extended to gather information from partially annotated proteins; see Supporting Information (Text S1, C). The inclusion of partially annotated proteins allows more protein pairs to be predicted and more neighbours to be included. Here we compare the prediction using only fully annotated proteins and all (fully and partially annotated) proteins.

The accuracy is the fraction of correct prediction out of all predictions against each of the 300 reference sets. Again, the 300 reference sets are employed to avoid the bias raised from too many negative pairs, i.e. a high accuracy may arise simply from making no positive prediction.

**Figure 7:**
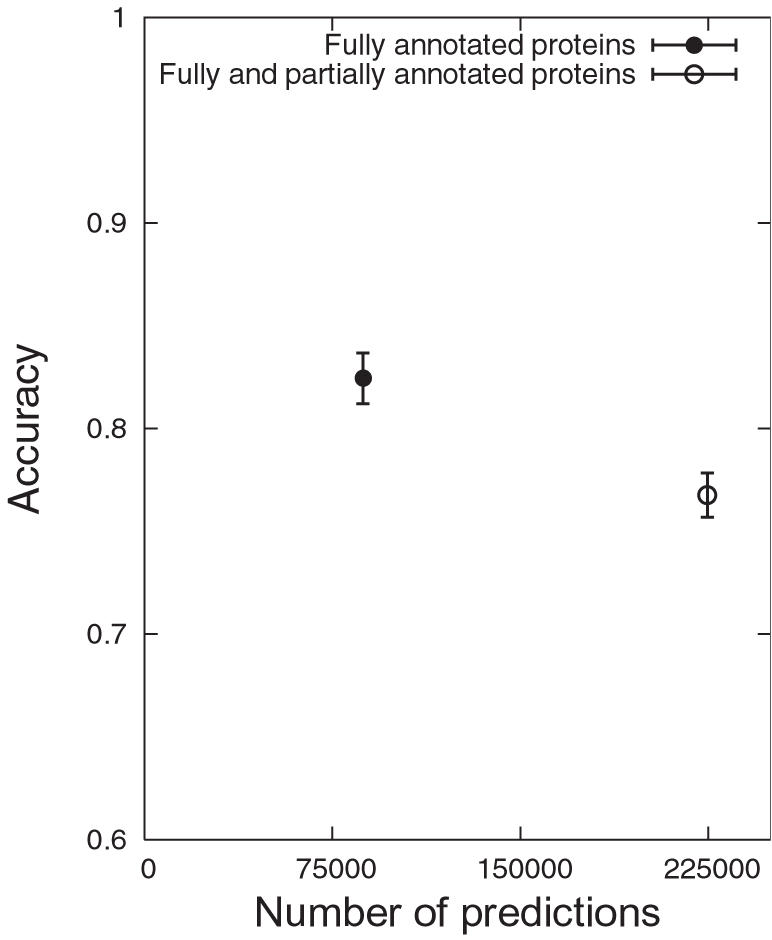
Accuracy and Coverage. Comparison of the number of predictions and accuracy between using, firstly, fully annotated proteins and secondly, fully and partially annotated proteins; the accuracy is the fraction of correct predictions out of all predictions against the reference set and is presented by an error bar (mean±2*standard deviation).

Figure 7 shows the accuracy and the coverage using fully or partially annotated proteins. The inclusion of partially annotated proteins considerably improves the coverage by 158% with an accuracy of 77% (only a drop of 5% from using fully annotated proteins).

### Using Different Prior Data Bases

To explore how different priors affect the prediction, we group protein interactions into prokaryotes, including *E.coli* and *H.pylori*, and eukaryotes, including *C.elegans*, *S.cerevisiae*, *D.melanogaster*, *M.musculus* and *H.sapiens*, and a final global pooled dataset including all interactions. As a random background, we also generate a simulated interaction network by shuffling the annotation of proteins in the Yeast protein interaction network. Based on the five prior data bases - Yeast, eukaryotes, prokaryotes, all interactions, and a shuffled protein network, we predict protein interactions using the triangle rate score. The AUC for all curves are calculated and tested for differences, see Table 5 and Text S1 and Table S5).

**Table 5:** AUC Based on Different Priors.

Predictive scores	Mean (*x̅*)	Sample standard deviation (*s*_x̅_)
Yeast	0.893	0.0058
Eukaryotes	0.874	0.0066
Prokaryotes	0.492	0.0119
All interactions	0.863	0.0067
Shuffled protein network	0.467	0.0088

**Figure 8:**
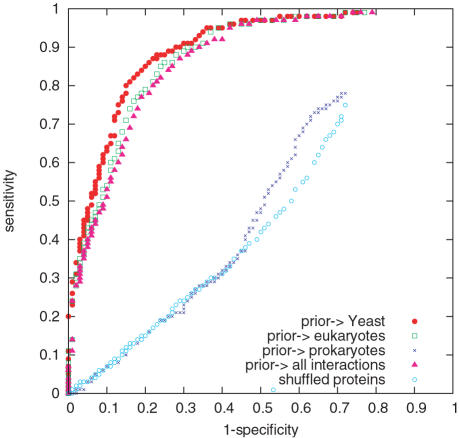
Performance by Using Different Prior Data Bases. ROC curves for the triangle rate score using upcast sets constructed, firstly, from yeast only, secondly, from all eukaryotes, thirdly, from all prokaryotes, and lastly, from all organisms. Randomly shuffled proteins are added for comparison.

The ROC curves show that the prior from Yeast itself gives the best prediction, followed by that from eukaryotes before third, all interactions; see Figure 8. The prior from prokaryotes gives almost no useful information, suggesting a fundamental difference of protein interaction networks between the two kingdoms. The difference between Yeast and eukaryotes probably arises because Yeast already has a large amount of interaction data, so that the inclusion of data from other similar organisms does not improve prediction. A less well studied organism however may benefit from a larger prior constructed from other close organisms. It is also not a surprise that the prior from eukaryotes performs slightly better, though not significantly, than the prior from all interactions, as the interactions from eukaryotes form the majority of interactions in the pool.

The clearly different ROC curves from the eukaryotes prior and the prokaryotes prior suggest that their networks are very different, in terms of the interaction patterns of protein characteristics. We perform a *χ* ^2^ test of homogeneity for triangles and lines in the two prior data bases. We compare characteristic triangles and lines that are annotated with structure, function and both, and group patterns with counts of at least 5. All 6 tests suggest a significant difference between eukaryotes and prokaryotes. This difference might arise from evolution and suggests that only priors from close organisms (within same kingdom) are helpful. It is not always beneficial to construct a large data base without taking the difference among organisms into account.

The ROC curves for predicting interactions from shuffled protein network are close to diagonal, as is expected. Without the information from protein structure and function and the interacting patterns, the prediction is random. The different trends between using real data and simulated data show that the interacting patterns of protein structure and function play important roles in protein interactions.

**Figure 9:**
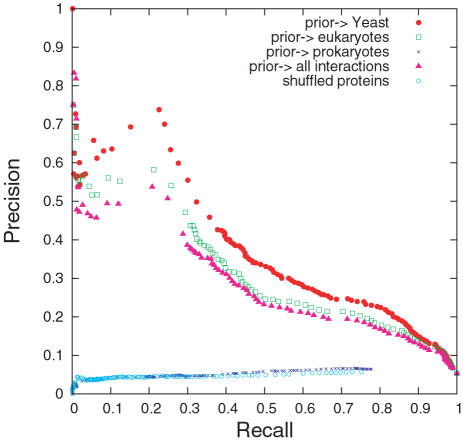
P-ROC Curves for Different Priors.

The P-ROC curve in Figure 9 shows a similar pattern in performance for the priors Yeast, eukaryotes, and all interactions, but it also reveals that taking prokaryotes as prior is worse than random shuffling. The figure shows that prokaryotes as prior could lead to perverse retrieval.

The different performance of prokaryotic and eukaryotic priors relates to their networks being rather different with respect to their distributions of protein structure and also of protein function. The most striking difference relates to small proteins. While 15% of eukaryote proteins are small proteins, less than 1% of prokaryote proteins are small proteins. Among the 10 most frequently observed structure category interactions, in eukaryote 3 of them (23% of all category interactions) involve small proteins, while in the list of top 10 structure category interactions in prokaryotes small protein related interactions do not appear. Another considerable difference concerns the distributions of the two functions “RNA polymerase II transcription factor activity” and “GTPase regulator activity”. While 4% of the eukaryotic proteins possess one of these two functions, they are not found in the prokaryotic proteins. In addition, in the list of top 10 most frequently observed function category interactions, in eukaryotic networks we observe many function category interactions with “protein binding” proteins, while they do not appear on the list of prokaryotes networks.

### Conclusion

With the triangle rate score we provide a novel statistical tool for prediction and validation of protein interactions. Our method uses triadic-level statistics, in addition to the traditional dyadic-level statistics arising pairwise interactions. This network-based method is shown to complement the existing domain-based approach, and to outperform the homology-based methods as well as a comparable pair-based method.

As our method requires annotated proteins occurring interacting with at least two other proteins, currently the only data set which is large enough to warrant application is that of Yeast, see G in Text S1 and also see Table S6; we anticipate that once more data will become available for many other organisms, our method will be useful in these organisms also.

Combining our method with priors from other organisms allows us to compare protein interaction behaviour among kingdoms, from the viewpoint of comparative interactomics. The significant difference in protein interactions networks between eukaryotes and prokaryotes serves not only as a caution to integrate interaction information from only close organisms, but also as encouragement for further, micro-level study between the two upcast sets, hoping for more insight into the biological difference between two kingdoms.

## Supporting Information

Text S1 Supporting Information Text
(1.13 MB DOC)

Table S1 Estimates of r-bar from characteristic triplets
(0.04 MB DOC)

Table S2 Observed patterns vs. all possible patterns†
(0.06 MB DOC)

Table S3 List of 7 classes in SCOP
(0.03 MB DOC)

Table S4 List of 24 main functional groups
(0.04 MB DOC)

Table S5 Z-tests for AUC comparison from the triangle rate score with different priors
(0.03 MB DOC)

Table S6 Numbers of protein interactions
(0.05 MB DOC)

Dataset S1 The list of 652 protein pairs, with score among our top 1%, but not in DIP
(0.01 MB TXT)

Dataset S2 The list of 49 protein pairs, with score among our bottom 5%, but which are found in DIP
(0.00 MB TXT)
