# Supplementary material for: Predicting and Validating Protein Interactions Using Network Structure
Source: PLoS Comput Biol. 2008 Jul 25;4(7):e1000118. doi: 10.1371/journal.pcbi.1000118 (PMC2435280; doi:10.1371/journal.pcbi.1000118)
Supplement: Text S1 — Supporting Information Text (1.13 MB DOC) [file pcbi.1000118.s001.doc]

**Supporting Information**

**A Tendency of formation of triangles**

Let $a$, $b$, $c$ $\in S$ be three characteristic vectors, and let $N$ be the set of proteins in the protein interaction network. Assume that all of $a$, $b$ and $c$ are indeed observed in the proteins, so that

and

For each type of category-category pair $\left\{ a,c \right\}$ with a fixed category $b$, the ratio of conditional probabilities is then estimated by

where

’

and

.

is the proportion of pairs of proteins $x,y$ in $N$, with characteristics such that $a\in S(x),b\in S(y)$, which interact, relative to all pairs of proteins with such characteristics. Note that, in contrast to the triangle rate score, we sum over proteins and keep characteristics fixed. Similarly, is the proportion of protein triplets, with given characteristics, which form a triangle, and is the proportion of protein triplets, with given characteristics, which form a line (but not a triangle).

For each organism (protein interaction network), $\bar{r}$ is the average of $\hat{r}_{\text{abc}}$ for all $a,b,c\in S$,

$$\bar{r}=\frac{\sum_{a,b,c\in S} \hat{r}_{abc}}{\frac{1}{2}|S|^{2}(|S|+1)}.$$

If , the existence of the interacting partner tends to decrease the chance of interaction. If , the interaction is more likely if two protein have an common interacting partner. The average ratios of conditional probabilities from different organisms are estimated in Table S1.

The standard errors (S.E.) in Table S1 are much larger in the estimates based on enhanced lines and triangles than in non-enhanced ones. The reasons may be that, firstly, many types of triples have only a few counts, (see Table S2), and secondly, missing data are likely to lead to biological triangles counted as lines.

False positive interactions may also contribute. In Table S1 all averaged ratios $\bar{r}$ exceed 1. This may suggest a tendency of forming triangles.

Table S2 is the numbers of observed types of triangles, lines and pairs in the upcast sets.

Table S1 Estimates of $\bar{r}$ from characteristic triplets

| Oganisms | #obs. pairs† | #obs. triples‡ | $\bar{r}$ | S.E. | $\bar{r}$ *>* 1§ |
| --- | --- | --- | --- | --- | --- |
| triangles/lines (structure) |  |  |  |  |  |
| D.M. | 23 | 94 | 5.6 | 2.76 | * |
| S.C. | 26 | 157 | 25.2 | 8.28 | * |
| E.C. | 20 | 105 | 9.6 | 4.14 | * |
| H.S. | 19 | 74 | 26.7 | 20.44 |  |
| triangles/lines (function) |  |  |  |  |  |
| D.M. | 110 | 534 | 48.3 | 67.6 |  |
| S.C. | 214 | 1850 | 55.9 | 91.36 |  |
| E.C. | 76 | 494 | 16.1 | 9.91 | *** |
| H.S. | 60 | 350 | 76.8 | 125.25 |  |

† number of different pairs {*a, c*} forming triples {*a~b~c*}

‡ total number of different triples {*a~b~c*}

§ 5% level of significance

*** organism showing tendency of formation of triangles

Table S2: Observed patterns vs. all possible patterns^†^

| Organisms | No. Triangles | % | All† | No. Lines | % | All | No. Pairs | % | All |
| --- | --- | --- | --- | --- | --- | --- | --- | --- | --- |
| enhanced triangles/lines/pairs (structure, function) | | | | | | | | | |
| D.M. | 1,045 | 0.10% | 804,440 | 83,793 | 3.50% | 2,384,928 | 2,980 | 21.00% | 14,196 |
| C.E. | 47 | 0.00% | 804,440 | 4,504 | 0.20% | 2,384,928 | 471 | 3.30% | 14,196 |
| S.C. | 11,701 | 1.50% | 804,440 | 131,124 | 5.50% | 2,384,928 | 3,588 | 25.30% | 14,196 |
| E.C. | 2,320 | 0.30% | 804,440 | 21,635 | 0.90% | 2,384,928 | 1,048 | 7.40% | 14,196 |
| M.M. | 6 | 0.00% | 804,440 | 766 | 0.00% | 2,384,928 | 267 | 1.90% | 14,196 |
| H.S. | 1,292 | 0.20% | 804,440 | 15,735 | 0.70% | 2,384,928 | 1,013 | 7.10% | 14,196 |
| non-enhanced triangles/lines/pairs (structure) | | | | | | | | | |
| D.M. | 42 | 50.00% | 84 | 192 | 98.00% | 196 | 28 | 100.00% | 28 |
| C.E. | 18 | 21.40% | 84 | 129 | 65.80% | 196 | 25 | 89.30% | 28 |
| S.C. | 68 | 81.00% | 84 | 186 | 94.90% | 196 | 28 | 100.00% | 28 |
| E.C. | 45 | 53.60% | 84 | 124 | 63.30% | 196 | 24 | 85.70% | 28 |
| M.M. | 2 | 2.40% | 84 | 56 | 28.60% | 196 | 16 | 57.10% | 28 |
| H.S. | 35 | 41.70% | 84 | 123 | 62.80% | 196 | 24 | 85.70% | 28 |
| non-enhanced triangles/lines/pairs (function) | | | | | | | | | |
| D.M. | 200 | 7.70% | 2,600 | 3,381 | 47.00% | 7,200 | 232 | 77.30% | 300 |
| C.E. | 9 | 0.30% | 2,600 | 367 | 5.10% | 7,200 | 74 | 24.70% | 300 |
| S.C. | 699 | 26.90% | 2,600 | 4,457 | 61.90% | 7,200 | 266 | 88.70% | 300 |
| E.C. | 187 | 7.20% | 2,600 | 1,200 | 16.70% | 7,200 | 136 | 45.30% | 300 |
| M.M. | 3 | 0.10% | 2,600 | 108 | 1.50% | 7,200 | 55 | 18.30% | 300 |
| H.S. | 139 | 5.30% | 2,600 | 730 | 10.10% | 7,200 | 102 | 34.00% | 300 |

†: The number of all possible patterns of triangles/lines/pairs, given the classification(s); for example, the total number of patterns of non-enhanced structural triangles is 84, in which three characteristic vectors are from one (7*1*1 possibilities), two (7*6*1 possibilities) or three (7!*/*3! possibilities) characteristic vectors.

**B Classifications of structure and function**

Table S3: List of 7 classes in SCOP

| Class | Description |
| --- | --- |
| *a* | All *α* proteins |
| *b* | All *β* proteins |
| *c* | Alpha and beta proteins (*α/β*), mainly parallel beta sheets (*β−α−β* units) |
| *d* | Alpha and beta proteins (*α* + *β*), mainly antiparallel beta sheets (segregated *α* and *β* regions) |
| *e* | *α* and *β*, folds consisting of two or more domains belonging to different classes |
| *f* | Membrane and cell surface proteins and peptides, not including proteins in the immune system |
| *g* | Small proteins, usually dominated by metal ligand, heme, and/or disulﬁde bridges |

† http://scop.mrc-lmb.cam.ac.uk/scop/data/scop.b.html

Table S4: List of 24 main functional groups

| GO id (ancient node) | Function |
| --- | --- |
| GO:0000166 | nucleotide binding |
| GO:0003676 | nucleic acid binding |
| GO:0003702 | RNA polymerase II transcription factor activity |
| GO:0003712 | transcription cofactor activity |
| GO:0004386 | helicase activity |
| GO:0005275 | amine transporter activity |
| GO:0005342 | organic acid transporter activity |
| GO:0005386 | carrier activity |
| GO:0005478 | intracellular transporter activity |
| GO:0005515 | protein binding |
| GO:0008047 | enzyme activator activity |
| GO:0008135 | translation factor ”activity,” nucleic acid binding |
| GO:0015075 | ion transporter activity |
| GO:0015144 | carbohydrate transporter activity |
| GO:0016491 | oxidoreductase activity |
| GO:0016564 | transcriptional repressor activity |
| GO:0016740 | transferase activity |
| GO:0016787 | hydrolase activity |
| GO:0016829 | lyase activity |
| GO:0016853 | isomerase activity |
| GO:0016874 | ligase activity |
| GO:0019207 | kinase regulator activity |
| GO:0030695 | GTPase regulator activity |
| GO:0043492 | ATPase activity |

Twenty-four frequently observed functional groups, selected from Molecular Function in Gene Ontology (http://www.geneontology.org/)

C Inclusion of partially annotated proteins

For a partially annotated proteins, *x* , the characteristic vectors in $S(x)$ is modified as in the example below in (C.1). If the annotation of $S_{m}$ for protein $x$ is unknown, vectors are generated by selecting each category in the characteristic $S_{m}$ (i.e., $s_{m}\in S_{m}$). As every category in $S_{m}$ is selected, the new vectors do not select any particular category and the information from $S_{m}$ serves purely as background.

Here we demonstrate the case when only two characteristics (e.g. structure and function) are used for building upcast sets and the triangle rate score. This concept can be generalized to the case of multiple characteristics.

The modified set of characteristic vectors for protein $x$ is now given by

$S(x)=\left\{\left.\begin{matrix} \left\{\left[s_{1}, s_{2}\right]\right. \\ \left\{\left[s_{1}, s_{2}\right]\right. \\ \left\{\left[s_{1}, s_{2}\right]\right. \end{matrix}\right|\begin{matrix} \left.s_{1}\in S_{1}(x), s_{2}\in S_{2}(x)\right\}, \text{if there is no missing annotation;} \\ \left.s_{1}\in S_{1}, s_{2}\in S_{2}(x)\right\}, \text{if annotation }s_{1}\text{ is missing;} \\ \left.s_{1}\in S_{1}(x), s_{2}\in S_{2}\right\}, \text{if annotation }s_{2}\text{ is missing.} \end{matrix} \right.$ (C. 1)

Extended triangle rate score The triangle rate score defined in equation (1) requires the query protein pair $\left\{ x, y \right\}$ and the common neighbour $z$ to be annotated with multiple characteristics. Here, an extended version of triangle rate is provided which allows proteins $x$ , $y$ and $z$ to be partially annotated.

When a triangle is composed by category(ies) from partially annotated proteins, the frequency of the triangle is multiplied by the relative frequency of this category among all categories in the characteristic. The total frequency of all characteristic triangle around $\left\{ x, y \right\}$ , $t_{\text{xy}}$ , is given by

(C. 2)

and the total frequency of all characteristic lines $l_{\text{xy}}$ is given by

(C. 3)

where the weight function $g_{x, y, z}$ is given according to the annotation status of proteins $x$ , $y$ and $z$,


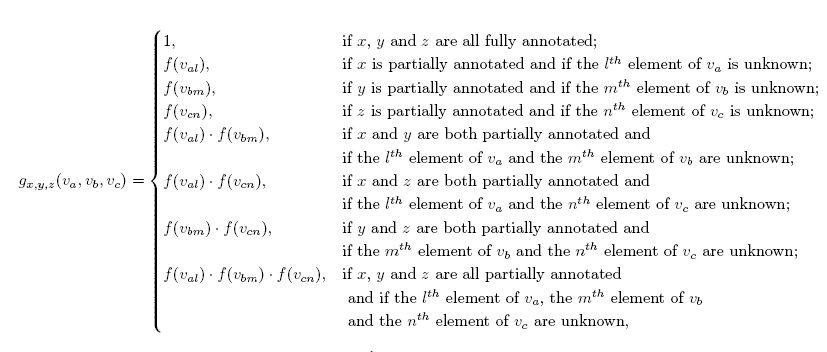


where $f(v_{\text{bm}})$ is the relative frequency of the $m^{\text{th}}$ element in vector $v_{b}$ . As we only consider two characteristics, there can be at most one missing annotation per vector. The triangle rate score can then be calculate from equation (1) with $t_{\text{xy}}$ and $l_{\text{xy}}$ obtained in (C. 2) and (C. 3).

Extended pair-based score The extended version of the pair-based score, based on the modified set of characteristic vectors for proteins (C. 1), is given by

The weight function $g_{x, y}$ is given according to the annotation status of proteins $x$ and $y$,

where $f(v_{\text{bm}})$ is the relative frequency of the $m^{\text{th}}$ element in vector $v_{b}$ . As we only consider two characteristics, there can be at most one missing annotation per vector.

**D Comparing two ROC curves**

Two ROC curves are compared through their areas under the curve (AUC). We calculate each AUC using the trapezoidal rule. The curve with larger AUC is regarded as a better curve, with higher accuracy and larger coverage, and therefore a better predictive score.

Let $x_{1}, x_{2},\cdots, x_{n}$ be $n$ AUC samples of the ROC curve **x** and $y_{1}, y_{2},\cdots, y_{m}$ be $m$ AUC samples of the ROC curve ***y*** . To see if the two mean AUC, $\mu_{x}$ and $\mu_{y}$ , are different, a two-sample *z* -test assuming unequal variances is employed for testing the hypothesis:

against

Let $\bar{x}$ and $\bar{y}$ be the average AUC of the two ROC curves. As $n$ is large, we assume that $\bar{x}$ follows a normal distribution , based on the central limit theorem, and similarly, we assume . The test statistic is given by

$$z=\frac{\bar{x}-\bar{y}}{\sigma_{\bar{x}-\bar{y}}},$$

where $\sigma_{\bar{x}-\bar{y}}$ is the standard deviation of area difference $(\bar{x}-\bar{y})$ . Under $H_{0}$ , the distribution of $z$ is approximately $N(0, 1)$.

Estimation In this paper, the prediction is verified against 300 reference sets, so that we have 300 samples of AUC per predictive method, i.e., $n=m=300$ . The 300 AUC are assumed to be independent.

**Mean area** The two mean areas $\mu_{x}$ and $\mu_{y}$ are estimated by the average values, $\bar{x}$ and $\bar{y}$ , of 300 AUC samples.

**Standard deviation** The standard deviation $\sigma_{\bar{x}-\bar{y}}$ is estimated by

$$s_{\bar{x}-\bar{y}}=\sqrt{s_{\bar{x}}^{2}+s_{\bar{y}}^{2}},$$

where $s_{\bar{x}}$ and $s_{\bar{y}}$ are the two sample standard deviations ().

When the null hypothesis is rejected at the 5%-level, we conclude that two predictive scores perform differently; one ROC curve with larger AUC is significantly better than the other one. Otherwise, the difference between two methods is not statistically significant.

E The logistic regression score

For each query protein pair, we also try to predict the interaction status by logistic regression using the triangle rate score as the explanatory variable and the binary interaction status as the outcome. In the logistic regression model, the probability of $x$ and $y$ to interact is given by

(E. 1)

where $a_{0}$ and $a_{1}$ are regression coefficients. Since protein pairs in the network may not occur independently, we employ a pseudo-likelihood approach to estimate $a_{0}$ and $a_{1}$ . This approach predicts one protein pair using parameters estimated from the rest of the network. Here we propose that our predictive score $S_{\text{xy}}$ for protein interactions is the estimated probability $\hat{p}_{\text{xy}}$ in the logistic regression model (E.1). We would therefore use

as an alternative score to predict and validate protein interactions.

In a nested model design, we also include both the pair-based score and the triangle rate score in the logistic regression. The probability of $x$ and $y$ having interaction is then modelled as

where $a_{0}$ , $a_{1}$ , and $a_{2}$ are the regression coefficients. Again the pseudo-likelihood approach is employed for coefficient estimation. The estimation of regression coefficients is based on training sets obtained by combining two equal-sized sets, one of observed interactions and one of unobserved interactions, as follows.

Estimation of logistic regression coefficients Here we describe how the training set for the estimation of regression coefficients is built for predicting one protein pair in a leave-one-out validation:

Let $G_{O}(V, N_{O})$ be the observed protein interaction network, where $V$ are the annotated proteins and $N_{O}$ are their observed interactions. Some eligible interactions among $V$ , not observed in $N_{O}$ , are denoted as $N_{U}$ . Because protein interaction networks are sparse, normally we have .

If the query protein pair $q$ is in $N_{O}$ , the network leaving out $q$ , , is the basis of our training set. If the query protein pair $q$ is in $N_{U}$ , the network is the basis of our training set. For all eligible interactions in $G_{T}$ , we calculate the pair score $\text{pair}(x, y)$ and the triangle rate score $\text{tri}(x, y)$ from the upcast set obtained from $G_{T}$.

Among these eligible interactions, we select $t$ sets of $m$ bootstrap samples from the observed interactions, $P_{i}$ ( $i=1,\cdots, t$ ), and $t$ sets of $m$ bootstrap samples from unobserved interactions, $N_{i}$ ( $i=1,\cdots, t$ ), where $m$ is the size of all observed interactions in $G_{T}$ . Therefore, the sample size for the estimation of the regression coefficients is $t$ . So, our $i$ -th training set is $T_{i}=P_{i}\cup N_{i}$ . The regression coefficients are calculated from every training set. Finally we use the average regression coefficients to estimate $a_{0}$ and $a_{1}$ (and $a_{2}$ ) in our logistic regression score.

Result Our preliminary investigation did not show significant improvement over the simple triangle rate score. The two ROC curves from the triangle rate score and the logistic regression score show very similar performance. The $z$ -test shows no difference between the mean AUCs. However, the full scale leave-one-out validation would be very computation-expensive. Since the triangle rate score is simpler and straightforward, we use it for the comparison and demonstration in this paper.

F Comparison of AUCs from different priors

Here are the results from using different priors in the triangle rate score. The z-tests show significant difference of AUCs from different priors except between eukaryotes and all interactions, and between prokaryotes and the shuffled protein network.

Table S5: Z-tests for AUC comparison from the triangle rate score with different priors

| Priors | Y | E | P | A | S |
| --- | --- | --- | --- | --- | --- |
| Yeast (Y) |  | 0.031 | *** | *** | *** |
| Eukaryotes (E) |  |  | * | 0.244 | *** |
| Prokaryotes (P) |  |  |  | *** | 0.091 |
| All interactions (A) |  |  |  |  | *** |
| Shuffled protein network (S) |  |  |  |  |  |

*** : *z*-score *>* 3.29, i.e., *p*-value *<* 0.001.

G Numbers of proteins for which our method is applicable

The number of protein interactions in various organisms, and the number of protein pairs which for which our method could currently be applied. When requiring fully annotated proteins, only S.cerevisiae has enough pairs to warrant application of the method.

Table S6: Numbers of protein interactions

| Organism | Annotation | Experimentally derived protein interactions | Eligible protein pairs | Eligible and observed protein pairs |
| --- | --- | --- | --- | --- |
| H.pylori | F | 1,420 | 103 | 5 |
|  | F+P |  | 3,803 | 59 |
| E.coli | F | 6,966 | 3,763 | 226 |
|  | F+P |  | 80,991 | 3,902 |
| C.elegans | F | 4,030 | 1,008 | 17 |
|  | F+P |  | 26,469 | 234 |
| D.melanogaster | F | 22,819 | 30,458 | 318 |
|  | F+P |  | 160,789 | 1,253 |
| H.sapiens | F | 1,397 | 2,338 | 199 |
|  | F+P |  | 4,563 | 313 |
| M.musculus | F | 290 | 102 | 3 |
|  | F+P |  | 319 | 20 |
| S.cerevisiae | F | 17,471 | 87,181 | 2,896 |
|  | F+P |  | 225,670 | 6,276 |

*F* : fully annotated

*F* + *P* : fully and partially annotated.
