# Supplementary material for: Predicting and Validating Protein Interactions Using Network Structure
Source: PLoS Comput Biol. 2008 Jul 25;4(7):e1000118. doi: 10.1371/journal.pcbi.1000118 (PMC2435280; doi:10.1371/journal.pcbi.1000118)
Supplement: Table S1 — Estimates of r-bar from characteristic triplets (0.04 MB DOC) [file pcbi.1000118.s002.doc]

| Oganisms | #obs. pairs† | #obs. triples‡ | $\bar{r}$ | S.E. | $\bar{r}$ *>* 1§ |
| --- | --- | --- | --- | --- | --- |
| triangles/lines (structure) |  |  |  |  |  |
| D.M. | 23 | 94 | 5.6 | 2.76 | * |
| S.C. | 26 | 157 | 25.2 | 8.28 | * |
| E.C. | 20 | 105 | 9.6 | 4.14 | * |
| H.S. | 19 | 74 | 26.7 | 20.44 |  |
| triangles/lines (function) |  |  |  |  |  |
| D.M. | 110 | 534 | 48.3 | 67.6 |  |
| S.C. | 214 | 1850 | 55.9 | 91.36 |  |
| E.C. | 76 | 494 | 16.1 | 9.91 | *** |
| H.S. | 60 | 350 | 76.8 | 125.25 |  |

† number of different pairs {*a, c*} forming triples {*a~b~c*}

‡ total number of different triples {*a~b~c*}

§ 5% level of significance

*** organism showing tendency of formation of triangles
