# Supplementary material for: Predicting and Validating Protein Interactions Using Network Structure
Source: PLoS Comput Biol. 2008 Jul 25;4(7):e1000118. doi: 10.1371/journal.pcbi.1000118 (PMC2435280; doi:10.1371/journal.pcbi.1000118)
Supplement: Table S2 — Observed patterns vs. all possible patterns† (0.06 MB DOC) [file pcbi.1000118.s003.doc]

| Organisms | No. Triangles | % | All† | No. Lines | % | All | No. Pairs | % | All |
| --- | --- | --- | --- | --- | --- | --- | --- | --- | --- |
| enhanced triangles/lines/pairs (structure, function) | | | | | | | | | |
| D.M. | 1,045 | 0.10% | 804,440 | 83,793 | 3.50% | 2,384,928 | 2,980 | 21.00% | 14,196 |
| C.E. | 47 | 0.00% | 804,440 | 4,504 | 0.20% | 2,384,928 | 471 | 3.30% | 14,196 |
| S.C. | 11,701 | 1.50% | 804,440 | 131,124 | 5.50% | 2,384,928 | 3,588 | 25.30% | 14,196 |
| E.C. | 2,320 | 0.30% | 804,440 | 21,635 | 0.90% | 2,384,928 | 1,048 | 7.40% | 14,196 |
| M.M. | 6 | 0.00% | 804,440 | 766 | 0.00% | 2,384,928 | 267 | 1.90% | 14,196 |
| H.S. | 1,292 | 0.20% | 804,440 | 15,735 | 0.70% | 2,384,928 | 1,013 | 7.10% | 14,196 |
| non-enhanced triangles/lines/pairs (structure) | | | | | | | | | |
| D.M. | 42 | 50.00% | 84 | 192 | 98.00% | 196 | 28 | 100.00% | 28 |
| C.E. | 18 | 21.40% | 84 | 129 | 65.80% | 196 | 25 | 89.30% | 28 |
| S.C. | 68 | 81.00% | 84 | 186 | 94.90% | 196 | 28 | 100.00% | 28 |
| E.C. | 45 | 53.60% | 84 | 124 | 63.30% | 196 | 24 | 85.70% | 28 |
| M.M. | 2 | 2.40% | 84 | 56 | 28.60% | 196 | 16 | 57.10% | 28 |
| H.S. | 35 | 41.70% | 84 | 123 | 62.80% | 196 | 24 | 85.70% | 28 |
| non-enhanced triangles/lines/pairs (function) | | | | | | | | | |
| D.M. | 200 | 7.70% | 2,600 | 3,381 | 47.00% | 7,200 | 232 | 77.30% | 300 |
| C.E. | 9 | 0.30% | 2,600 | 367 | 5.10% | 7,200 | 74 | 24.70% | 300 |
| S.C. | 699 | 26.90% | 2,600 | 4,457 | 61.90% | 7,200 | 266 | 88.70% | 300 |
| E.C. | 187 | 7.20% | 2,600 | 1,200 | 16.70% | 7,200 | 136 | 45.30% | 300 |
| M.M. | 3 | 0.10% | 2,600 | 108 | 1.50% | 7,200 | 55 | 18.30% | 300 |
| H.S. | 139 | 5.30% | 2,600 | 730 | 10.10% | 7,200 | 102 | 34.00% | 300 |

†: The number of all possible patterns of triangles/lines/pairs, given the classification(s); for example, the total number of patterns of non-enhanced structural triangles is 84, in which three characteristic vectors are from one (7*1*1 possibilities), two (7*6*1 possibilities) or three (7!*/*3! possibilities) characteristic vectors.
