# Supplementary material for: Predicting and Validating Protein Interactions Using Network Structure
Source: PLoS Comput Biol. 2008 Jul 25;4(7):e1000118. doi: 10.1371/journal.pcbi.1000118 (PMC2435280; doi:10.1371/journal.pcbi.1000118)
Supplement: Table S3 — List of 7 classes in SCOP (0.03 MB DOC) [file pcbi.1000118.s004.doc]

| Class | Description |
| --- | --- |
| *a* | All *α* proteins |
| *b* | All *β* proteins |
| *c* | Alpha and beta proteins (*α/β*), mainly parallel beta sheets (*β−α−β* units) |
| *d* | Alpha and beta proteins (*α* + *β*), mainly antiparallel beta sheets (segregated *α* and *β* regions) |
| *e* | *α* and *β*, folds consisting of two or more domains belonging to different classes |
| *f* | Membrane and cell surface proteins and peptides, not including proteins in the immune system |
| *g* | Small proteins, usually dominated by metal ligand, heme, and/or disulﬁde bridges |

† http://scop.mrc-lmb.cam.ac.uk/scop/data/scop.b.html
