# Supplementary material for: Predicting and Validating Protein Interactions Using Network Structure
Source: PLoS Comput Biol. 2008 Jul 25;4(7):e1000118. doi: 10.1371/journal.pcbi.1000118 (PMC2435280; doi:10.1371/journal.pcbi.1000118)
Supplement: Table S4 — List of 24 main functional groups (0.04 MB DOC) [file pcbi.1000118.s005.doc]

| GO id (ancient node) | Function |
| --- | --- |
| GO:0000166 | nucleotide binding |
| GO:0003676 | nucleic acid binding |
| GO:0003702 | RNA polymerase II transcription factor activity |
| GO:0003712 | transcription cofactor activity |
| GO:0004386 | helicase activity |
| GO:0005275 | amine transporter activity |
| GO:0005342 | organic acid transporter activity |
| GO:0005386 | carrier activity |
| GO:0005478 | intracellular transporter activity |
| GO:0005515 | protein binding |
| GO:0008047 | enzyme activator activity |
| GO:0008135 | translation factor ”activity,” nucleic acid binding |
| GO:0015075 | ion transporter activity |
| GO:0015144 | carbohydrate transporter activity |
| GO:0016491 | oxidoreductase activity |
| GO:0016564 | transcriptional repressor activity |
| GO:0016740 | transferase activity |
| GO:0016787 | hydrolase activity |
| GO:0016829 | lyase activity |
| GO:0016853 | isomerase activity |
| GO:0016874 | ligase activity |
| GO:0019207 | kinase regulator activity |
| GO:0030695 | GTPase regulator activity |
| GO:0043492 | ATPase activity |

Twenty-four frequently observed functional groups, selected from Molecular Function in Gene Ontology (http://www.geneontology.org/)
