# Supplementary material for: Predicting and Validating Protein Interactions Using Network Structure
Source: PLoS Comput Biol. 2008 Jul 25;4(7):e1000118. doi: 10.1371/journal.pcbi.1000118 (PMC2435280; doi:10.1371/journal.pcbi.1000118)
Supplement: Table S5 — Z-tests for AUC comparison from the triangle rate score with different priors (0.03 MB DOC) [file pcbi.1000118.s006.doc]

| Priors | Y | E | P | A | S |
| --- | --- | --- | --- | --- | --- |
| Yeast (Y) |  | 0.031 | *** | *** | *** |
| Eukaryotes (E) |  |  | * | 0.244 | *** |
| Prokaryotes (P) |  |  |  | *** | 0.091 |
| All interactions (A) |  |  |  |  | *** |
| Shuffled protein network (S) |  |  |  |  |  |

*** : *z*-score *>* 3.29, i.e., *p*-value *<* 0.001.
