# Supplementary material for: Predicting and Validating Protein Interactions Using Network Structure
Source: PLoS Comput Biol. 2008 Jul 25;4(7):e1000118. doi: 10.1371/journal.pcbi.1000118 (PMC2435280; doi:10.1371/journal.pcbi.1000118)
Supplement: Table S6 — Numbers of protein interactions (0.05 MB DOC) [file pcbi.1000118.s007.doc]

| Organism | Annotation | Experimentally derived protein interactions | Eligible protein pairs | Eligible and observed protein pairs |
| --- | --- | --- | --- | --- |
| H.pylori | F | 1,420 | 103 | 5 |
|  | F+P |  | 3,803 | 59 |
| E.coli | F | 6,966 | 3,763 | 226 |
|  | F+P |  | 80,991 | 3,902 |
| C.elegans | F | 4,030 | 1,008 | 17 |
|  | F+P |  | 26,469 | 234 |
| D.melanogaster | F | 22,819 | 30,458 | 318 |
|  | F+P |  | 160,789 | 1,253 |
| H.sapiens | F | 1,397 | 2,338 | 199 |
|  | F+P |  | 4,563 | 313 |
| M.musculus | F | 290 | 102 | 3 |
|  | F+P |  | 319 | 20 |
| S.cerevisiae | F | 17,471 | 87,181 | 2,896 |
|  | F+P |  | 225,670 | 6,276 |

*F* : fully annotated

*F* + *P* : fully and partially annotated.
